# Parents and Caregivers Satisfaction After Palliative Treatment of Spastic Hip Dislocation in Cerebral Palsy

**DOI:** 10.3389/fneur.2021.635894

**Published:** 2021-03-18

**Authors:** Aleksander Koch, Joanna Krasny, Magdalena Dziurda, Magdalena Ratajczyk, Marek Jozwiak

**Affiliations:** Department of Pediatrics Orthopedics and Traumatology, University of Medical Sciences, Poznan, Poland

**Keywords:** pain, spastic hip dislocation, salvage procedures, cerebral palsy, palliative treatment

## Abstract

**Objectives:** Pain appearance is one the most common complication of spastic hip disease in children with cerebral palsy (CP). It determines child and caregiver quality of life and life priorities. Reconstruction hip surgery should be considered as a treatment of choice. Some clinical conditions give the inability to perform such a procedure. In our paper, we would like to present four palliative methods of spastic hip dislocation treatment in children with CP.

**Material:** We analyzed four groups of patients treated because of hip pain. Inclusion criteria were pain appearance (visual analog scale-11 or numeric rating scale-11) and hip joint dislocation (migration percentage >80%). All patients were admitted to our department between 2008 and 2018. In the first group, patients were treated only by steroid injections to hip joints; in the second group, patients were recruits after hip interposition arthroplasty with shoulder spacer; in the third group, they were patients after valgus subtrochanteric osteotomy (Schanz); and in the fourth group, these were patients after proximal femoral resection (Castle procedure). The minimal follow-up time was 2 years. The first group consisted of 15 patients (15 hips) with a mean age of 15.5 (8–17) years; the second group, 20 patients (24 hips) with a mean age of 14.2 (9–22.6) years; the third group, 22 patients (24 hips) with a mean age of 13.5 (7–20.5) years; and the fourth group, 10 patients (15 hips) with a mean age of 12.9 (7–17.6) years.

**Methods:** Radiological evaluation was based on a standardized anteroposterior X-ray of the hip joints. Pain severity before surgery and at the last follow-up time was measured by visual analog scale-11. Parents or caregivers were asked about their child's pain during sitting, perineal care, and rest. During the visit, all caregivers were asked about treatment satisfaction (no 0 to max 10) and if they would decide again for the same surgery.

**Results:** In all groups of patients, we observed a decrease in pain complaints. The observed reduction of pain in the first group was from 7.88 (4–10) to 3.08 (0–8) (*p* = 0.05), but results of injection were observed only for 4 months (2–8), and it has to be repeated (average: two times). In the second group, level of pain was reduced from 4.93 (1–10) to 0.93 (0–5) (*p* < 0.001); in the third group, from 6.22 (3–10) to 0.59 (0–6) (*p* < 0.001); and in the fourth group, pain reduces from 5.43 (2–10) to 2.13 (0–5) (*p* < 0.001). Observed changes concerned mostly sitting position and perineal care. The complication rate was in the second group, 6 of 24 cases of extraarticular ossification; in the third group, 2 of 24 cases with extraarticular ossification, two cases of revision surgery. In the fourth group, two cases needed another femoral resection. In the first group, five patients died during follow-up time, so they were excluded from the study. In the steroid injection group, parents' treatment evaluation was 6.83 (0–10), and only in three cases that they would resign from the treatment. In the hip interposition arthroplasty group, caregivers' evaluation was 7.41 (0–10), and in five cases, parents did not accept the surgery. In the Schanz osteotomy group, parents' evaluation was 5.9 (0–10), and in eight cases, caregivers would not repeat surgery. In the proximal femoral resection group, satisfaction was the highest, 8.3 (3–10), and only two parents would not decide for surgery again.

**Conclusion:** All procedures can be considered as palliative treatment options for pain complain in a spastic hip joint dislocation in children with CP. Steroid injections to the hip joint need to be repeated, and with the follow-up time, it becomes less effective. Steroid injection seems to be the treatment of choice for patients with general anesthesia contraindications. Interposition arthroplasty of the hip joint seems to give better final results, but the highest parents' satisfaction surprisingly was observed in the proximal femoral resection group, but differences were not statistically significant.

## Introduction

Spastic hip dislocation is the second most common orthopedic manifestation of cerebral palsy (CP) after equinus deformity of the foot (1). All the hip pathologies potentially found in children with CP are collectively described as a spastic hip disease (SHD) (2). The severity of this medical issue is strongly connected with patients' functional level described by the Gross Motor Function Classification System (GMFCS) (3). Because one of the most severe complications of SHD is a complete hip dislocation (migration percentage >80%), the classification system also provides a prognostic value. The hip subluxation in GMFCS level I is reported to be below 1%, whereas GMFCS levels IV and V have a subluxation risk of 70–90%, defined as migration percentage above 33% (4). Hip dislocation among non-ambulators can lead to chronic pain, sitting imbalance, pelvic obliquity, and scoliosis (1, 5–8). Parents or caregivers often complain about problems with hygiene and perineal care. The medical professionals should regard “Proreactive” soft tissue release or “reactive” hip reconstruction procedures such as open reduction and femoral varus derotation with shortening osteotomy or pelvic Dega osteotomy as the treatment of choice (9, 10). When the struggle to prevent hip dislocation is lost, chronic pain and permanent dislocation can be managed with palliative salvage procedures such as Schanz osteotomy (SO), Girdlestone, Castle resection, McHale procedure, or finally proximal femur prosthetic interposition arthroplasty (PFIA) (11–14). There exist many papers describing salvage treatment options for children with CP, but the literature is quite scant in comparative studies (15–17). Our main interest is to find an optimal treatment method for chronic painful spastic hip dislocation. Thus, the purpose of this study is to compare parents' or caregivers' satisfaction with treatment and to assess the treatment results in terms of reported pain due to spastic hip dislocation.

## Methods and Operative Technique

A retrospective analysis of all patients with painful hip dislocation treated in our department in the years 2008 to 2018 was performed. We identified a total of 312 patients, of which 167 had palliative treatment (primary or secondary). To this group, we applied inclusion criteria such as diagnosis of CP, GMFCS level IV or V, hip pain, and at least 2 years of follow-up. Excluding cases with any previous hip reconstruction procedures has yielded a total of 67 patients who fall under one of the four therapeutic groups: steroid injections (SIs), Castle hip resection (HR), Schanz femoral valgus osteotomy (SO), and PFIA. All groups are described in Table 1.

**Table 1:** Description of the groups.

	**Steroid injection group (SI)**	**Femoral head resection group (HR)**	**Shanz osteotomy group (SO)**	**Proximal femoral interposition arthroplasty group (PIFA)**
Patients/Hip Joints	15/15	10/15	22/24	20/24
Age	15.5 years (8–17)	12.9 years (7–17)	13.5 years (7–20)	14.2 years (9–22)
GMFCS IV Patients/Hip Joints	0	0	8/9	2/3
GMFCS V Patients/Hip Joints	15/15	10/15	14/15	18/21
Male/Female	9 (9 hips)/6 (6 hips)	5 (8 hips) – 5 (7 hips)	14 (15 hip)/8 (9 hips)	12 (14 hips) – 8 (10 hips)

The pain was assessed before surgery and at the last follow-up either with a self-report (providing sufficient child's communication skills) or with a parent or caregiver report, which was the case most often. The pain severity was recorded during the child's rest (lying position), perineal care, and sitting using a visual analog scale (VAS)-11 or numeric rating scale (NRS)-11, depending on the child's communication skills. The satisfaction of parents/caregivers with treatment was also recorded at each follow-up using a modified VAS—Satisfaction or NRS-11 (no 0 – max 10). Also, parents were surveyed whether given a choice again they would have decided for the same surgical treatment option once again on the operating site. Statistical analysis was performed using Statistica 12 and PQ stat software. To assess the statistical differences between groups, the Student's *t*-test and chi-square test were used to compare two groups, and analysis of variance was used when comparing more than two groups. Correlation between parameters was evaluated by Pearson's correlation coefficient (rs). Differences were considered significant with *p* < 0.05. Due to the selected group of patients and collected data, the choices of statistical tests were fully approved and correct.

The SI group included patients who could not be operated (general medical condition, no consent for the proposed treatment) and obtained at least 2 SIs (Betamethasone), minimum 3 months apart. The SI was performed during contrast arthrography in the operation theater under fluoroscopic guidance. The HR group underwent a proximal femoral resection at the level of the ischial tuberosity according to Castle procedure with the femoral end cap made of bone cement. The acetabulum was covered by a double-layered hip joint capsule. The post-surgery hip traction for 2–4 weeks was applied to every patient. The SO group underwent a subtrochanteric osteotomy, according to Shanz, with an average value of valgus correction of 60°. The primary decision behind all cases from the SO group was to perform an inspection of the hip. Only the intraoperative evaluation of the femoral head shape with the joint cartilage was providing grounds for SO in place of hip reconstruction. The osteotomy was stabilized with locking or a blade plate without cast immobilization but with the use of abduction splint post-surgery.

**Figure 1:**
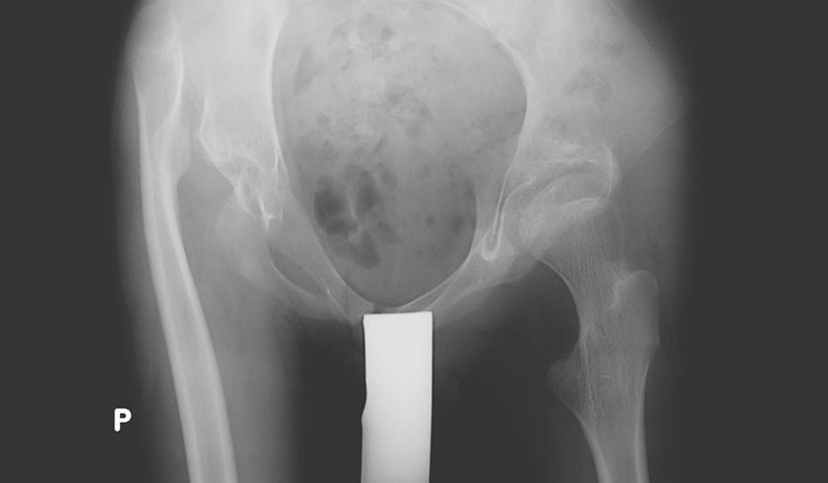
17 y old, male, treated because painful hip dislocation.

**Figure 2:**
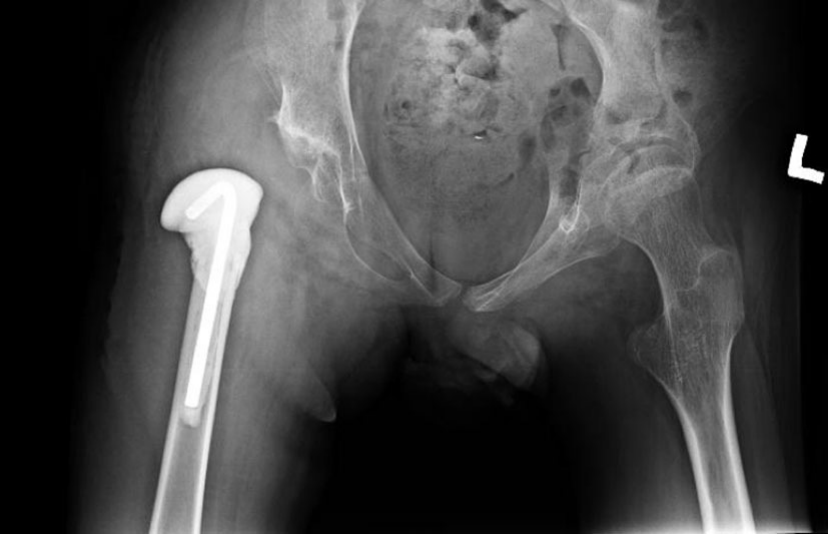
Patient after PFIA – right side.

Regarding PFIA, the final decisions were taken before surgery. The resection was performed under the same level as in Castle procedure, but to protect soft tissues and acetabulum, we used a temporary shoulder spacer Tecres^®^ Spacer-S, which was fixed with bone cement. This implant has the smallest endoprosthesis stem that can be used (Figures 1 and 2). Furthermore, the size of the stem can be further slimmed to fit the femoral intramedullary canal. Before spacer implantation, two holes are drilled in the proximal end of the femur for better and more stable fixation of the spacer in the femur with the bone cement. During surgery, almost a complete resection of the joint capsule is performed. The acetabulum is covered by the double-layered fold sutured from fascia and muscles, i.e., gluteal, adductors, and iliopsoas.

## Results

**Figure 3:**
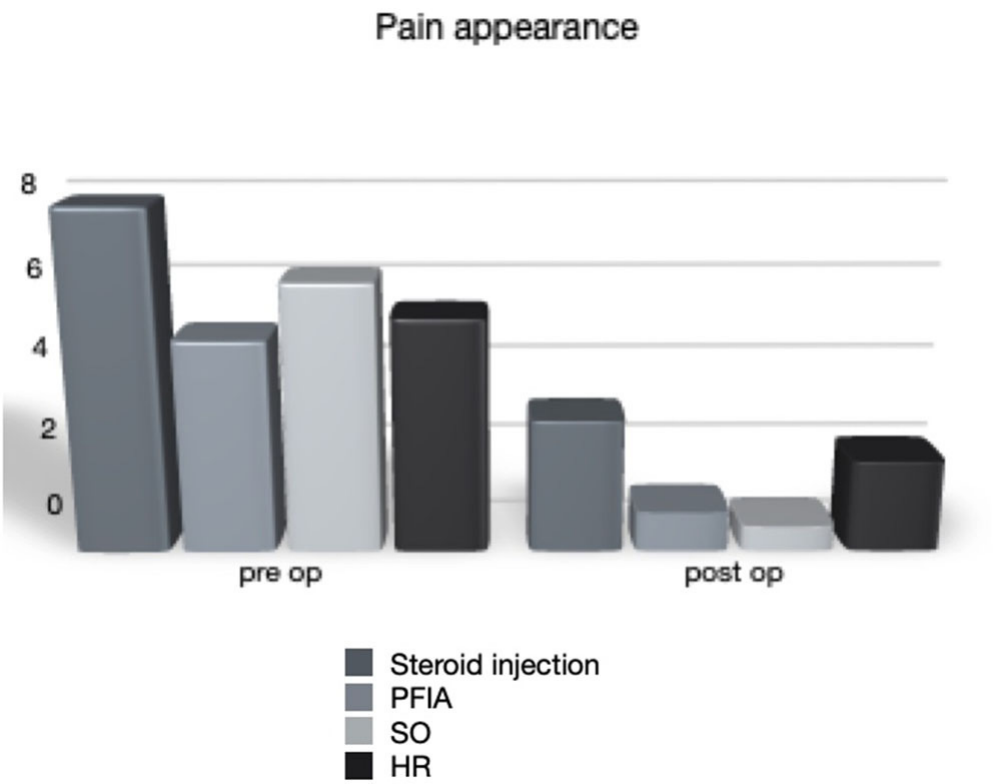
Pain appearance before and after the treatment.

A decline in pain complaints in all four therapeutic groups was observed. The average pain in the SI group declined from 7.88 (range = 4–10) to 3.08 (range = 0–8) (*p* < 0.001). The analgesic effect was observed for an average of 4 months (range = 2–8), and the treatment was repeated at least once (twice on average). The PFIA, SO, and HR groups recorded a pain relief from 4.93 (1–10) to 0.93 (0–5), 6.22 (3–10) to 0.59 (0–6), and 5.42 (2–10) to 2.13 (0–5) in the NRS score, respectively (*p* < 0.001; Figure 3). The pain score was reduced comparably in all the three activities during which it was recorded, i.e., lying position, perineal care, and sitting position. The reduction difference was statistically significant in all cases except patients after proximal femoral resection, where the change in pain at the lying position was not statistically significant (*p* = 0.15). There were no differences between patients that were GMFCS levels IV and V in the SO or the PIFA groups.

The extent of pain relief correlates strongly with age, i.e., the older the patient, the smaller the reduction in pain, at sitting (*p* = 0.04, *rs* = −0.24) and perineal hygiene (*p* = 0.01, *rs* = −0.29) in particular.

**Figure 4:**
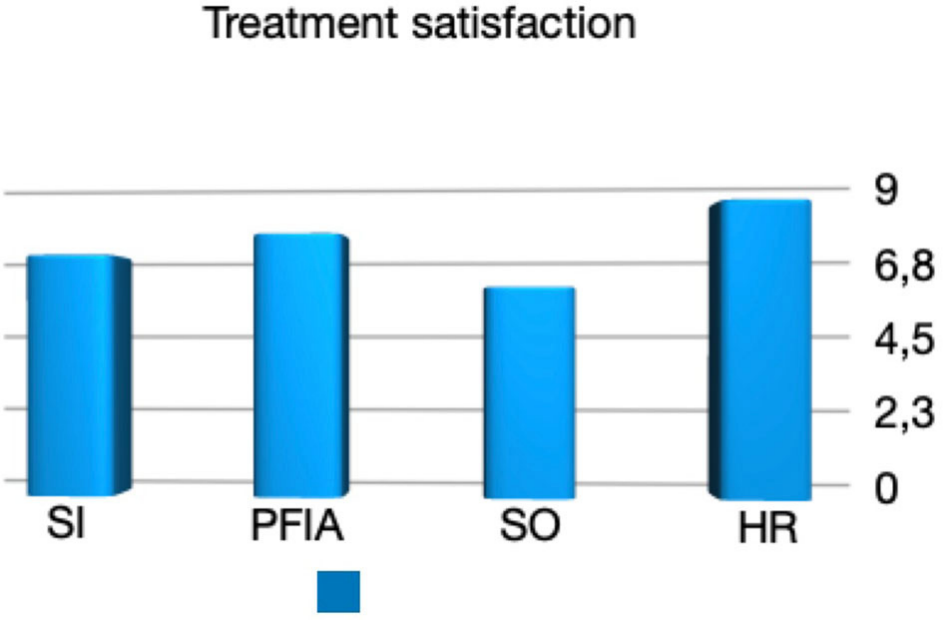
Parents or Caregivers treatment satisfaction.

The treatment satisfaction scores reported by the parents or caregivers were 6.83 (0–10), 7.41 (0–10), 5.9 (0–10), and 8.3 (3–10) for the SI, PFIA, SO, and HR groups, respectively (Figures 1, 3). The proportions of parents or caregivers who would not have consented to the surgery again were 3/15, 5/20, 8/22, and 2/10, respectively (Figure 4).

**Figure 5:**
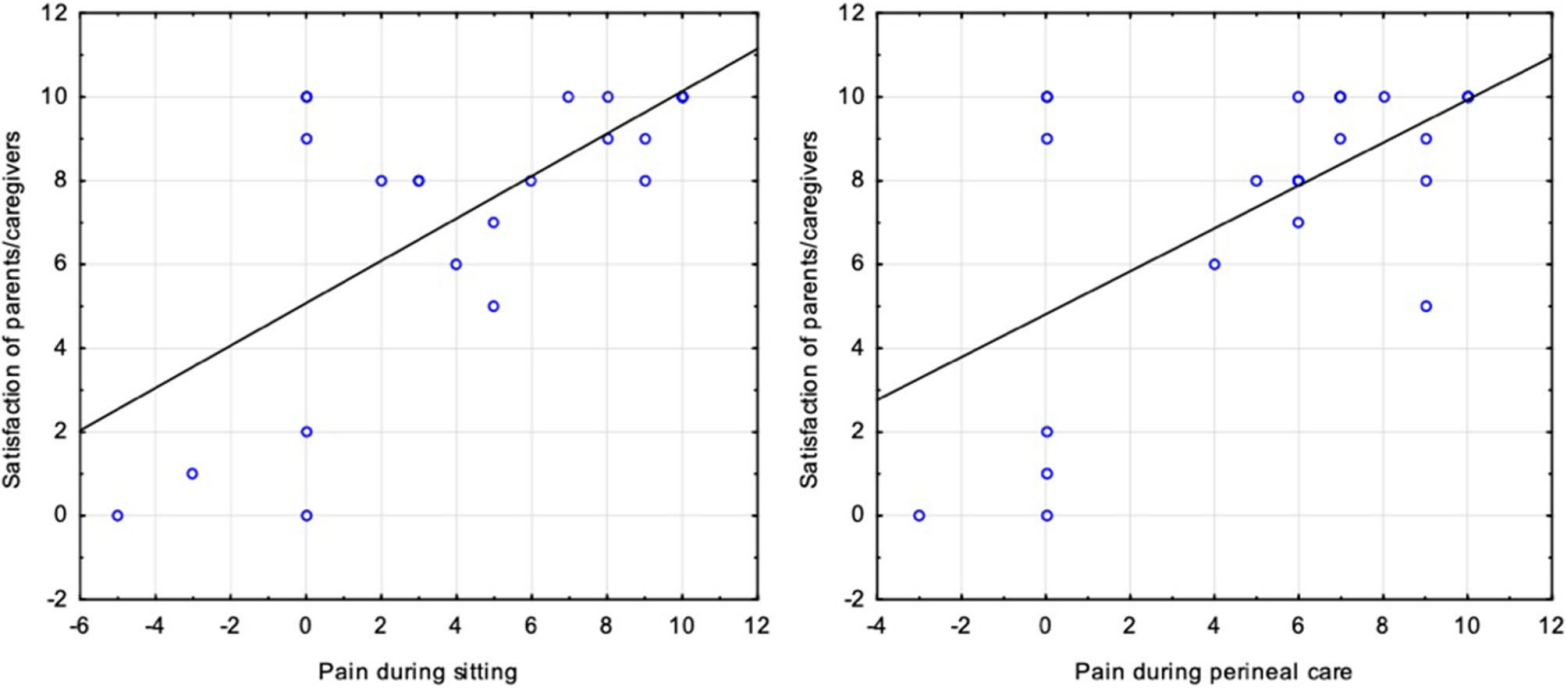
Correlation between Pain and Parents satisfaction.

Expectedly, the extent of pain relief correlates with the degree of satisfaction of parents with the treatment. Patients after PFIA had a significant pain difference during all three activities (lying, perineal care, and sitting) that correlated well with the parent/caregiver satisfaction (respectively: *p* = 0.002, *rs* = 0.58; *p* = 0.01, *rs* = 0.49; *p* = 0.001, *rs* = 0.62; Figures 2, 5). By the same token, the decrease of pain was strongly associated with the parents' acceptance expressed by the willingness to repeat the surgical treatment, especially for the group of patients after PFIA (*p* = 0.004).

In the SI group, five patients have died during the follow-up period, resulting in exclusion from the study. In the PFIA group, a total of six hips had a quite typical complication of extraarticular ossification in contrast to only two hips after Schanz valgus osteotomy that shared the same sequela. Also, two patients required revision surgery in both the SO and HR groups because of hardware failure and femoral stump migration, respectively.

## Discussion

Global pain complaints can be observed in 22–75% of non-ambulatory children with CP with great neurological impairment in particular. Children with GMFCS levels IV and V are at the highest risk of developing painful progressive hip displacement. The natural history of SHD shows that only 50% of dislocated hip joints are painful. Why the rest is pain-free remains elusive (1, 18). Because communication problems are often seen in such patients, parents, or caregivers who take care of a severely handicapped child are in the best position to observe and interpret the child's behavioral or idiosyncratic signals in reaction to pain. Indeed, many authors focus mainly on the parent-reported pain suffered by the child as a proper source of information (19–21).

Hip reconstruction surgery should always be considered as a treatment of choice for SHD (10). This procedure includes soft tissue release, derotation varus shortening femoral osteotomy, and transiliac pelvic osteotomy providing no degenerative cartilage changes. Non-ambulatory patients with a chronic spastic hip dislocation who develop pain due to joint cartilage lesion should be offered a rather palliative treatment (15, 16, 20, 22–25). Among salvage treatment options, there is no preferred surgical procedure. Many are described, including proximal femoral resection, valgus osteotomy, McHale procedure, PFIA, and total hip replacement (26, 27). It has been reported that almost all salvage hip procedures may bring pain relief, and they are almost equally effective at reducing the level of pain (15, 25). Some considerations have to be listed when a decision about surgery is to be taken. Proximal femoral resection is a relatively short and simple procedure, but patients will require traction treatment, and the lower extremity will lose its weight-bearing potential (11, 17, 19, 23). Femoral proximal stump migration is a consequence often seen in a proximal femoral resection. Heterotropic ossification and hardware failure are the most commonly observed problems after Schantz valgus osteotomy, but patients retain the possibility for assisted standing with partial weight-bearing after surgery (24, 28).

Total hip replacement is an option for ambulatory CP patients, but complex bony deformities, severe osteoporosis, and a pelvic obliquity may constitute a relative contraindication. In CP patients with GMFCS levels IV and V, the total hip endoprosthesis is rather avoided (15, 26, 27).

Hip arthrodesis is a method that is least recommended for its high rate of post-operative complications. It was proved that children with CP after hip fusion had impaired bone healing and discomfort in regular life due to a constrained hip position (15).

According to Sliverio et al., PFIA is a preferred salvage option for painful spastic hip dislocation in children with CP whose conditions improved after surgery. The risk connected with PFIA is acceptable and comparable with the other salvage procedures (21). The results of our study lend support to PFIA being widely accepted by the parents and bringing a well-documented patient improvement. Patel et al. pointed that proximal femoral resection provides a pain-free movable articulation (19). However, during longer follow-up time, symptoms may recur and will probably be connected with proximal femoral end migration. Patel suggests that an interposition myopathy may function as a mechanical barrier, which helps to stop the migration of the proximal femur and allow for articulation with the iliac wing (19). Furthermore, PFIA is regarded as the best palliative treatment option in the paper of Wright et al. (20). Also, there is a consensus of all authors suggesting that post-operative traction treatment is only needed after proximal femoral resection (15, 19). Chan et al. compared femoral resection, valgus osteotomy, and arthroplasty in light of patient ability for weight-bearing. They assumed that patients and parents are willing to maintain the possibility to weight-bearing post-operatively if they were able to do it preoperatively. They declined the procedure of proximal femoral resection (22). Godfrey described a modified McHale procedure and believes it is the most effective and efficient operative treatment technique for painful SHD (23). In contrast to pointing out a single procedure, Kolman et al. recommend all palliative hip procedures except hip arthrodesis, which has a very high rate of post-operative problems (16). They also point out that every palliative procedure has a potential complication: femoral head resection—femur migration; heterotypic ossification and Valgus osteotomy—problems with implants; and PFIA—revision surgery and fracture of long bones. Interestingly, there are no papers describing SI as a method of hip pain treatment, in contrast with the common use of this method in clinical practice. Our study shows a decrease in pain after hip joint SI. It has time-limited effectiveness (2–6 months) but may be suitable for painful non-operative patients. In our opinion, it is the first paper describing the effectiveness of serial hip intraarticular SIs in children with SHD, and on the basis of our material, we can recommend this treatment option for this selective group of patients.

Our study shows that the decision about surgery in chronic spastic hip dislocation is not straightforward. Steroid injection to the hip joint is generally a simple procedure but needs to be repeated. It seems to be the treatment of choice for patients with short life expectancy or with general contraindications. Interposition arthroplasty seems to give better long-term results than other procedures. Surprisingly, the highest parents' satisfaction was observed in the proximal femoral resection group, but the differences were not statistically significant (*p* > 0.05). Based on the literature and our experience, we recommend PFIA as a surgical treatment for chronic spastic painful hip dislocation in children with CP with GMFCS levels IV and V. The optimal salvage procedure for GMFCS level IV remains undefined, as PIFA has a potential cost of losing the ability for assisted standing.

One fact that we could not find in other papers was a correlation between the age of the patient and the level of pain complaints. Older patients are more willing to have severe pain complaints. Probably, it is due to a period because the child has a diagnosis of spastic hip dislocation, and finally, a decision about surgery was made. Duration of dislocation may lead to cartilage lesions and sensitization of pain receptors around the hip joint, which is obviously caused by direct and indirect inflammatory factors (18). It also implicates the final result of the treatment because older patients get a smaller reduction of pain after applied treatment.

Reduction of pain is the main goal of chronic spastic hip dislocation treatment. Many authors had proved that palliative treatment leads to a decrease in pain complaints. Very often, it is only described if the pain was or not before and after surgery (20–22). Patel et al., in a paper describing proximal femoral resection, get a reduction of pain from 7.8 to 2.8 according to VAS (19). Our results for that group of patients were comparable, from 5.42 to 2.13. Results for the other three groups are similar to the other researchers (15, 16, 19–24).

## Study Limitation

Because patients did not get preventive or reconstructive hip surgery, their general health condition and communication skills were quite poor and non-homogenous in pain status. Consequently, most of the pain assessments were parent/caregiver reports in place of self-reports, which results in all sorts of potential bias and limitations to comparison. The experience of our orthopedic team is based on more than 50% of all cases treated because of the neuromuscular condition, but presented results are not statistically significant, so we cannot recommend one easy and simple solution for chronic painful spastic hip dislocation.

## Conclusion

All procedures presented in our paper can be considered as palliative treatment options for chronic painful spastic hip dislocation in children with CP. Steroid injection to the hip joint is a generally simple procedure but needs to be repeated. Steroid injection seems to be the treatment of choice for patients with general anesthesia contraindications. Interposition arthroplasty of the hip joint seems to give better final results, but differences were not statistically significant (*P* > 0.05). On the basis of the literature and our experience, we recommend PFIA as a surgical treatment of chronic spastic painful hip dislocation in children with CP with GMFCS level V.

## Data Availability Statement

The raw data supporting the conclusions of this article will be made available by the authors, without undue reservation.

## Ethics Statement

The studies involving human participants were reviewed and approved by Bioethical Committee University of Medical Sciences in Poznan, Poland. Written informed consent to participate in this study was provided by the participants' legal guardian/next of kin.

## Author Contributions

AK, JK, MD, MR, and MJ contributed to the design and implementation of the research, to the analysis of the results, and to the writing of the manuscript. All authors contributed to the article and approved the submitted version.

## Conflict of Interest

The authors declare that the research was conducted in the absence of any commercial or financial relationships that could be construed as a potential conflict of interest.
